# Mycobacterium abscessus Causes Highly Resistant Infection as a Breast Abscess

**DOI:** 10.7759/cureus.38450

**Published:** 2023-05-02

**Authors:** John M Coggins, Ann Obi, Joel Badders, Khushali Roy, Rachel Duncan, Colleen Silva

**Affiliations:** 1 Surgery, University of Texas Medical Branch at Galveston, Galveston, USA

**Keywords:** rapidly growing mycobacterium, mycobacterium abscessus, antimicrobial resistance, abscess, breast

## Abstract

*Mycobacterium abscessus* is an increasing threat to public health due to its multi-drug resistance and increasing prevalence. The pathogen most commonly causes chronic respiratory infections, but it may also invade locally through the skin and soft tissue damage caused by trauma, piercings, or tattoos. A 58-year-old African American female presented with a five-month history of recurrent abscesses in the right breast. She had previously been treated with doxycycline and ceftriaxone injections at an outside clinic with minimal improvement. Following incision and drainage, cultures and susceptibilities showed *M. abscessus* infection with a high level of drug resistance. Due to financial barriers, the patient received a suboptimal antibiotic regimen and required multiple surgical procedures, resulting in only temporary wound healing. At the time of this report, the patient is recovering from her fourth incision and drainage, with cultures and susceptibilities pending and discussions of a total mastectomy. *M. abscessus* is a highly resistant bacteria capable of causing skin and soft tissue infections of the breast. Such infections may occur without an inciting event and require extensive surgical and antimicrobial management.

## Introduction

*Mycobacterium abscessus* is a rapidly growing mycobacterium that is amply found in soil and water [1]. It is comfortable in a variety of settings, having been isolated from hot tubs, livestock, environmental dust, endoscopes, and water systems [2,3]. The ubiquity of *M. abscessus* is owed to its ability to withstand high levels of chlorine, form biofilms, and resist high temperatures [4]. Patients with preexisting lung diseases, particularly cystic fibrosis, are at an increased risk of becoming infected, making *M. abscessus* the most common cause of nontuberculous mycobacterial (NTM) lung disease [5]. The pathogen may also cause skin and soft tissue infections, whose incidences are increasing [6]. Immunodeficiencies and uncontrolled diabetes mellitus are risk factors, and procedures like piercings and tattoos may be related to such infections as the bacteria are resistant to disinfectants [1,7].

The diagnosis of an *M. abscessus* infection is made by culture with susceptibilities and isolation of the specific subspecies if possible [8]. Three subspecies have been identified: abscessus, bolletii, and massiliense [9]. Infection of the skin or soft tissue typically requires surgical debridement followed by extensive antibiotic therapy for a minimum of 6-12 months. *M. abscessus* is heavily resistant to pharmacotherapy at baseline and poses a significant challenge to treat. The choice of antibiotics is greatly dependent upon the infection’s susceptibility to macrolides, as they are the mainstay of treatment. Subspecies abscessus and bolletii are commonly resistant to macrolides, and cure rates range from 30-50% [8]. Subspecies' massiliense is generally susceptible to macrolides, and cure rates range from 80-90% [10]. Therapeutic regimens are highly variable as they should be tailored to the susceptibility testing for each infection and are regularly changing as research advances our understanding of these dangerous infections. Generally, second-generation macrolides, third-generation tetracyclines, amikacin, cefoxitin, and clofazimine have been the most utilized antibiotics, as they have had the best in vitro antimicrobial activity and generally have low rates of resistance [11]. If debridement followed by antibiotic therapy is unsuccessful, clinicians may repeat the process or eventually consider more aggressive surgical procedures, as in the patient to be presented.

## Case presentation

**Figure 1:**
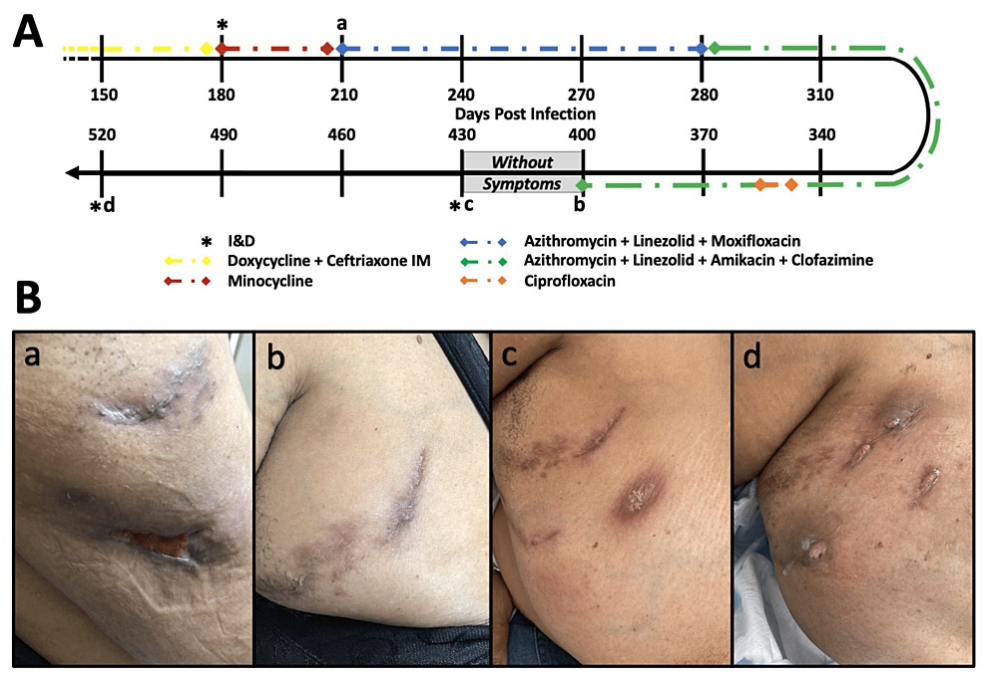
Breast abscess timeline (A) A timeline of the patient’s infection with Days Post Infection indicating the number of days after the patient first noticed the breast lesions. Letters a,b,c,d indicate images taken in clinic of the lesions and shown in part B of the figure. Asterisks indicate instances incision and drainage (I&D) was performed. Colored dashed lines indicate antibiotic regimens administered at the time. The grey box indicates a period of time during which the patient was without symptoms; at all other times illustrated the patient was symptomatic. (B) Images a,b,c,d show the right breast lesions with part A of the figure indicating the timepoint of infection. Image a was taken 30 days after presentation to clinic and 180 days after lesions first presented, at which time the patient had received three ceftriaxone shots and doxycycline at an outside clinic. Image b was taken after initiating treatment with linezolid, azithromycin, amikacin, and clofazimine. Image c shows the recurring abscess which presented after one month of being symptom free without antibiotic therapy. Image d was taken three months later and shows five actively draining lesions.

A 58-year-old African American female with a past medical history of poorly controlled diabetes mellitus and hypertension presented to the clinic with a five-month history of painful, spontaneously draining lesions in the upper outer quadrant of the right breast. She reported a recent history of similar-appearing lesions on the left upper abdomen that resolved spontaneously. The patient reported no known exposure to trauma, soil, or water as a possible source of infection, nor had the patient undergone a procedure, tattoo, or piercing of the surrounding skin. The patient was postmenopausal and had previously breastfed her two children. She reported no history of breast-related conditions, infections, or abscesses. A routine mammogram obtained one year prior to presentation was Breast Imaging-Reporting and Data System (BI-RADS) 1 bilaterally. She reported previously receiving ceftriaxone injections with mild improvement and was currently taking doxycycline by mouth when the patient presented to the breast surgery clinic. She reported symptoms of fever and chills and severe pain and drainage from the lesions. A physical exam revealed two large abscesses in the upper outer quadrant of the breast actively draining pus (Figure 1). The abscesses were incised and drained in the clinic, and cultures were obtained. Two days later, the surrounding skin remained erythematous, the abscesses continued draining purulent fluid, and the patient reported worsening pain. The patient was taken to the operating room for a repeat incision and drainage (I&D) and discharged on minocycline with no follow-up complications and pending cultures.

After two weeks, cultures grew *M. abscessus*, and pathology showed acute and chronic granulomatous inflammation with giant cell reactions. As the patient’s symptoms were stable, the patient was continued on minocycline while waiting for drug susceptibilities. Two weeks later, the patient presented with worsening pain and erythema surrounding the abscesses, indicating the progression of the infection. The patient was started on linezolid, azithromycin, and moxifloxacin, with susceptibilities still pending. Shortly after, the susceptibility results showed resistance to moxifloxacin and intermediate resistance to linezolid, with azithromycin still pending (Table 1). The patient was recommended a peripherally inserted central catheter (PICC) line placement and a treatment regimen of clofazimine, imipenem, eravacycline, and amikacin. Unfortunately, the patient had social and financial barriers to this treatment regimen, and the antibiotic regimen was modified to include clofazimine, linezolid, azithromycin, and amikacin.

**Table 1:** Antibiotic susceptibilities Cultures of *M. abscessus* were tested for antibiotic susceptibilities following the first incision and drainage of the breast abscesses. Antibiotics were scored as susceptible, intermediate, or resistant based on the minimum inhibitory concentration (MIC) using Clinical and Laboratory Standards Institute (CLSI) guidelines for nontuberculous mycobacteria (NTM) [12]. *Clofazimine and Bedaquiline have not received CLSI breakpoints for NTM infections.

Antibiotic	Susceptibility	MIC (µg/ml)
Clarithromycin	Susceptible	2
Linezolid	Intermediate	16
Tigecycline	Intermediate	0.12
Amikacin	Intermediate	32
Cefoxitin	Intermediate	64
Azithromycin	Intermediate	≥8
Clofazimine	*	0.12
Bedaquiline	*	0.12
Moxifloxacin	Resistant	≥16
Ciprofloxacin	Resistant	≥8
Doxycycline	Resistant	≥32
Minocycline	Resistant	≥16
Imipenem	Resistant	64

The patient received a PICC line and was started on clofazimine 100 mg daily, azithromycin 500 mg daily, linezolid 600 mg twice daily, and amikacin 1540 mg (22 mg/kg for an ideal body weight of 70 kg) twice weekly with an aim of 12 weeks of treatment. Clofazimine was initiated as a Single-Patient Initiated New Drug (SPIND) approved drug. The patient was monitored with a weekly complete blood count (CBC) and comprehensive metabolic panel (CMP), an amikacin trough before each treatment, and daily saline flushes of the PICC line. During this time, the breast abscess continued to heal and showed no signs of a progressing infection. After nine weeks of therapy, the patient developed leukocytosis and fever due to Serratia marcescens bacteremia. The PICC line was replaced, and the patient received a seven-day course of ciprofloxacin. After four months of antibiotic therapy, the abscesses resolved completely, and all antibiotic therapy was discontinued.

**Figure 2:**
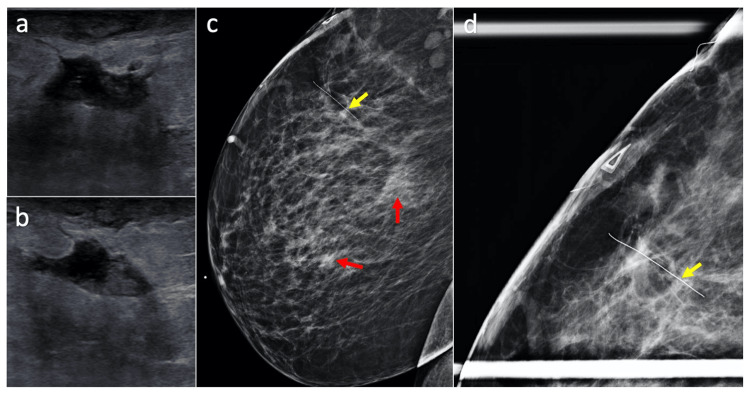
Imaging of breast abscess Images A and B are perpendicular ultrasound views of an 18 × 17 × 8 mm^3^ hypoechoic mass in the right breast at 11 o'clock, 13 cm from the nipple at an area of palpable concern. Signs of mild skin thickening and trabecular thickening are present. Images C and D show tomosynthesis imaging of the right breast with a mediolateral view and signs of scattered fibroglandular density present (red arrows). Image d includes compression of the area of palpable concern demarcated by the wire (yellow arrows).

One month after discontinuing antibiotics, the patient developed a new breast abscess in the same location, which was imaged with ultrasound and tomosynthesis (Figure 2). An I&D was conducted in the operating room, and staining showed a few acid-fast bacilli, but tissue cultures grew no organisms. Antibiotics were not prescribed. Three months later, the patient presented with five breast abscesses, which were painful and draining. The abscesses were excised completely and sent for pathology and microbiology. The patient had no post-operative complications at her one-week follow-up. Cultures returned positive for *M. abscessus*, and new susceptibilities are pending at the time of this report. Two months after the I&D, the abscesses resumed draining purulence. Given the extensive resistance of this pathogen, numerous I&D procedures, difficulties affording the appropriate medications, and reoccurrence of the abscesses after escalating antibiotic therapies, a total mastectomy is being considered and discussed with the patient.

## Discussion

Skin-penetrating trauma is the primary cause of skin and soft tissue infections with *M. abscessus*. Cosmetic procedures like breast implantation and liposuction or exposure during a piercing or tattoo are reported causes of infection [1,7,13]. This patient’s lack of exposure to *M. abscessus* by breach of the skin barrier is uncommon; however, the clinical presentation as local skin abscesses and the histological findings were consistent with a typical *M. abscessus* skin infection [13].

*M. abscessus* is well known for its ability to resist powerful antibiotics, making treatment difficult, costly, and prolonged. Second-generation macrolides are often efficacious, but a large portion of *M. abscessus* contains an erythromycin ribosome methylase (erm) gene, which confers resistance to macrolides [10]. The gene is rarely expressed in vitro until after 14 days of culture, requiring *M. abscessus* and other rapidly growing mycobacteria (RGM) to have extended incubation periods for proper MIC testing [14]. To hasten the process, clinicians may elect for genetic sequencing to determine if the pathogen is macrolide-sensitive. Genetic sequencing was not conducted in our case. Azithromycin and clarithromycin are generally regarded as equally effective against *M. abscessus* bacteria lacking the erm gene [15]. The specific macrolide chosen may be related to cost, possible drug interactions, or simply clinician preference [16]. However, there is evidence that azithromycin is more effective against the subspecies M. abscessus subsp. abscessus [16]. In light of this, azithromycin was chosen for our patient.

Treatment of *M. abscessus* with a macrolide alone is generally insufficient to fully treat the infection and may cause greater antibiotic resistance [13]. Most commonly, a macrolide is combined with a third-generation tetracycline, amikacin, cefoxitin, or clofazimine according to culture susceptibilities [13]. Accordingly, in this case, the patient was initially recommended eravacycline, amikacin, clofazimine, and imipenem. A macrolide was not included due to the intermediate resistance to macrolides seen with culture. Unfortunately, the patient was unable to afford the copay for this regimen, with eravacycline and imipenem increasing the patient’s copay the most. As a result of this, the patient received a suboptimal regimen, and the time between surgery and the initiation of pharmacotherapy was greatly lengthened due to the logistics of coordinating a new, affordable treatment plan.

Surgical treatment of *M. abscessus* skin and soft tissue infections typically involves incision and drainage of the abscess, debridement of necrotic tissue, and excision of infected tissue. In some cases, reconstructive surgery may be necessary to repair tissue damage caused by the infection. While surgical intervention has been shown to be effective in managing these infections, it is important to note that recurrence rates can be high, and several reports describe patients who required mastectomies due to infection recurrence [17].

## Conclusions

*M. abscessus* is a multi-drug-resistant NTM capable of causing skin and soft tissue infections that are extremely difficult to treat. Surgery followed by several months of a potent antibiotic regimen is recommended but is often insufficient to fully treat the infection. Infections of the breast are amenable to more aggressive surgical options.
